# Long-Term Trend Analysis in the *Solar Radiation and Climate Experiment* (SORCE)/*Spectral Irradiance Monitor* (SIM)

**DOI:** 10.1007/s11207-022-02001-9

**Published:** 2022-06-01

**Authors:** Jerald W. Harder, Stéphane Béland, Steven Penton, Thomas N. Woods

**Affiliations:** Laboratory for Atmospheric and Space Physics, University of Colorado, Boulder, CO 80303 USA

**Keywords:** Solar spectral irradiance, Sun-climate observations, Instrument effects

## Abstract

The *Solar Radiation and Climate Experiment*/*Spectral Irradiance Monitor* (SORCE/SIM) instrument was launched on 25 January 2003 with mission termination occurring on 25 February 2020. The SORCE/SIM provides a unique data set of the variability in solar spectral irradiance (SSI) during the descending phase of Solar Cycle 23 (SC23) from April 2003 to February 2009, the weaker solar-maximum conditions of SC24, and the quiescent SC24/SC25 minimum. The determination of the magnitude and phase of SSI variations rely on the unambiguous determination of the effects of the space environment and solar-exposure-related degradation mechanisms. The instrument-only corrections for SIM are based on a comparison of two functionally identical (mirror image) prism spectrometers with four independent detectors in each spectrometer channel. The degradation correction is strictly instrumental in its methodology and makes no assumptions about the magnitude, slope, or wavelength dependence of the SSI variability.

## Introduction

This article is the fourth in a series of articles published in *Solar Physics* related to the design, operation, calibration, and performance of the *Solar Irradiance Monitor* (SIM) onboard the *Solar Radiation and Climate Experiment* (SORCE) spacecraft. The first (Harder et al., 2005a) describes the scientific requirements, design, and operation modes for the instrument. The second (Harder et al., 2005b) discusses the fundamental measurement equations and the pre-flight-calibration methodology for the instrument. The third (Harder et al., 2010) continues the discussion of the absolute calibration of the instrument, describing additional post-launch characterizations using flight-spare components and comparisons with the SORCE and *Upper Atmosphere Research Satellite/SOlar Stellar Irradiance Comparison Experiment* (UARS)/SOLSTICE instruments and the *Atmospheric Laboratory of Applications and Science* (ATLAS) composite (Thuillier et al., 2004). Additional inflight comparisons with the European Space Agency *Environmental Satellite, SCanning Imaging Absorption spectroMeter for Atmospheric CHartographY* (ESA ENVISAT/SCIAMACHY) instrument are discussed by Pagaran et al. (2011). More recently, the *Total and Spectral Solar Irradiance Sensor* (TSIS-1) was deployed on the *International Space Station* on 15 December 2017. An intensive intercomparison of the two SIM instruments was conducted over the last two years of the SORCE mission during the Solar Cycle 24 minimum (Harder et al., 2022). Some initial comparisons between SORCE/SIM and TSIS-1/SIM are provided by Mauceri et al. (2020). Furthermore, Richard et al. (2020) and Mauceri et al. (2020) provide pre-flight and in-flight instrument characterizations and calibrations for TSIS-1/SIM, which can be compared to similar techniques for SORCE/SIM.

Harder et al. (2005a) provide a detailed description of the SIM instrument, but the basic optical configuration is briefly described here. There are two SIM channels called SIMA and SIMB: SIMA is used for daily solar measurements, and SIMB is used for tracking SIMA degradation trends and only makes solar observations about once a month. Each spectrometer channel has an entrance slit, a Féry prism, and a set of exit slits for each detector in its focal plane. The reference (stable) detector is an Electrical Substitution Radiometer (ESR) that is used for correcting the photodiodes and providing a data product for the 1600 – 2401.5 nm spectral region. There are three different photodiodes to cover the wide SIM spectral range of 240 nm to 1600 nm, and these photodiodes are referred to as the UV, VIS1, and IR photodiodes.

Over the course of its 6159 day (16.86 years) operational lifetime, SORCE/SIM produced 5815 spectra covering the 240 – 2401.5 nm wavelength range. The SORCE instruments and spacecraft were operational nearly 12 years beyond their five-year prime-mission lifetime. With the completion of the SORCE mission, a reassessment of the observed spectral irradiance is required, particularly in the light of additional systematic uncertainties imposed on the spectral-irradiance measurements due to the inevitable degradation of key spacecraft and instrument subsystems that first appeared in January 2008. As part of the final deliverables for the SORCE mission, NASA requested a revised Algorithm Theoretical Basis Document (ATBD) to refine data corrections that were made subsequent to launch. This was particularly important for the SIM instrument because it was the first instrument of its kind to be deployed in space. The SORCE/SIM ATBD is a publicly available document that provides extensive documentation of the instrument’s performance during the entire mission and is cited as Harder et al. (2021) in this article. The SIM ATBD can be downloaded via doi.org/10.25810/rff8-ff38.

In lieu of lengthy and detailed appendices, this article provides a summary of the degradation mechanisms and the methodology applied to correct the data. The SORCE/SIM data used for this article are Version 27 of SIM and are available both through LASP and the NASA Goddard Earth Sciences Data and Information Services Center (GES DISC): lasp.colorado.edu/home/sorce/data/ and disc.gsfc.nasa.gov/datasets?keywords=SOR3SIMD _027.

Important discussion also appears in the Version 27 release notes found at lasp.colorado.edu/home/sorce/instruments/sim/sorce-sim-data-products-release-notes/.

## Instrument-Degradation Measurement Equation

The measurement equation that determines the response of the instrument can be written with terms that isolate the sensitivity-loss mechanisms so they can be determined separately and then reintroduced into the equation in a physically meaningful way. Section 2.1 discusses contributions to the measurement equation appropriate for the SIM electrical substitution radiometer (ESR) and for the photodiode detectors, and Section 2.2 introduces the prism transmission-degradation function. Greater detail on how these contributions are analyzed appears in Sections 4 through 7 of this article. The reader is referred to Harder et al. (2005b) and Harder et al. (2010) for greater insight into the pre-flight measurement equations used to determine the irradiance of the Sun. The purpose of this article is to describe how on-orbit corrections to the pre-flight measurement equation are determined.

It is important to note that degradation proceeds both through clock time and through the length of time the instrument is exposed to solar radiation. These mechanisms must be determined and included separately in the overall degradation measurement equations along with their associated uncertainties. Table 1 gives a listing of the most significant contributions to degradation that are segregated into effects that do not accumulate with time, and those that accumulate with time and produce irreversible trends in the irradiance time series. The short-term effects in this table essentially specify the precision of a single measurement and therefore are critical for the determination of the long-term trends in the time series because they represent the ultimate limit that detectors and spectrometers can be compared.

**Table 1:** Sources of uncertainty in the SIM time series.

Source	Effect	Magnitude of effect	Mitigation
*Short-term effects – do not accumulate with time*			
Spacecraft pointing	Local perturbation in prism transmission, wavelength shift	Produces spurious noise of <1%	Irradiance vs. spacecraft pointing offset linear fit
Scattered light	Increases apparent irradiance, decreases contrast of solar structure	<100 ppm in ESR, VIS1, IR, < 0.5% in UV	No correction, invariant in time
Detector temperature	Spurious structure in photodiode data	See Section 4.3	Refined temperature coefficients
Prism temperature	Wavelength shift from temperature coefficient of refractive index	About 150 ppm (cf. Malitson, 1965)	Adequately corrected in processing
Detector noise	Ultimate limit of comparison for two spectra	See Section 4.4	No action
*Long term effects -accumulate with time*			
Wavelength shift/ alignment change	Discontinuous changes in instrument response and wavelength grid at well-defined times (at safe-hold events)	*λ* uncertainty ≈ (1.5×10^−3^) × FWHM resolution	Shift/stretch each spectrum in record
ESR servo gain degradation	Reduction in the responsivity of the ESR detectors	7 ppm yr^−1^,	Routine onboard gain calibration
		Uncertainty ≈ 1 ppm yr^−1^	
Trend differences between detectors	Degradation trends for photodiodes do not match the trends of the ESR	See Section 6.	Reconcile photodiode trend to ESR, correct photodiode radiant sensitivity
Prism-transmission degradation	Dominant irreversible reduction in instrument response	See Section 7.	SIMA & SIMB intercomparison and correction

### Detector-Specific Degradation

Solar spectral irradiance [$E$($\lambda ,t$)], as a function of wavelength [$\lambda $] and time [$t$] can be expressed in its most elementary form from the measured radiant power [$P(\lambda,T,t)$] of a detector divided by the profile integral [$S(\lambda ,T,t)$], noting that temperature [$T$] corrections must be included. Equation 1 shows the solar spectral irradiance (SSI) with units of W m^−2^ nm^−1^:

1 $$ E \left ( \lambda ,t \right ) = \frac{P \left ( \lambda ,T,t \right )}{S \left ( \lambda ,T,t \right )}. $$

The profile integral, Equation 2, combines the wavelength-dependent contributions from the spectrometer resolution and detector-wavelength dependencies that span the width of a spectral-resolution element and applies the needed corrections to the temperature-sensitive components:

2 $$ S \left ( \lambda _{s},T,t \right ) =A \left ( T \right ) \int \alpha ( \lambda ,T,t)\operatorname{Tr} \left ( \lambda ,T \right ) \Phi \left ( \lambda \right ) S ' \left ( \lambda _{s},\lambda \right ) \mathrm{d} \lambda , $$

**Table:** 

$S(\lambda _{\mathrm{S}},T,t)$	=	Profile integral at selected wavelength $\lambda _{\mathrm{S}}$ [m^2^ nm]
*T*	=	Temperature [°C]
*t*	=	Clock time on orbit [seconds]
*A*(*T*)	=	Entrance slit area [m^2^]
*α*(*λ*,*T*,*t*)	=	Detector specific sensitivity
Tr(*λ*,*T*)	=	Pre-flight transmission of the prism glass [unitless]
Φ(*λ*)	=	Diffraction correction for the entrance slit [unitless]
$\text{S}'(\lambda _{s}, \lambda )$	=	Full-width half-maximum of a spectroscopic resolution element at $\lambda _{\mathrm{S}}$ [nm].

The detector specific sensitivity [$\alpha $], shown in Equation 2, is different for the ESR (unitless) and photodiodes (units of Amperes per Watt); however, each measures the radiant power of light that passes through the entrance slit. The time dependence for the two detectors will be discussed shortly. Equation 2 does not show a time dependence for $\operatorname{Tr}(\lambda ,T)$, since that appears as the prism-degradation function discussed in Section 2.2 and expressed in Equation 5.

The ESR is a thermal detector that operates in a phase-sensitive detection mode. The reader is referred to Harder et al. (2005b), Section 4.1 for details of the ESR characteristics, notation, and calibration. In Equation 2, the term $\alpha $($\lambda $) is the reflectivity of the nickel-phosphorous black surface on the bolometer; it ranges in value from 0.9985 to 0.9995 as a function of wavelength and is not dependent on temperature. The power measurement for the ESR [$P$_ESR_($\lambda ,t$)] with units of Watts, can be written symbolically as shown in Equation 3:

3 $$ P_{\mathrm{ESR}} \left ( \lambda ,t \right ) = M_{\mathrm{ESR}}^{-1} V_{7}^{2} R_{\mathrm{VDR}} \left \{ \frac{1+ \tilde{G} \left ( t \right )}{\tilde{G} \left ( t \right )} \frac{\boldsymbol{p} \cdot \boldsymbol{D}}{\boldsymbol{p} \cdot \boldsymbol{Q}} \right \}, $$

**Table:** 

$P_{\mathit{ESR}}$(*λ*,*t*)	=	Measured ESR power [Watts]
*t*	=	Clock time on orbit [second]
M_ESR_	=	Scaling factor for data output [60,000 data numbers, unitless]
V_7_	=	Value of the 7.1 voltage reference [Volts]
R_VDR_	=	Voltage-divider ratio formed from bolometer heater and a series resistor [Ohms]
$\tilde{G} \left ( t \right )$	=	Open-loop gain of the detector amplifier as a function of time [unitless]
$\frac{\boldsymbol{p} \cdot \boldsymbol{D}}{\boldsymbol{p} \cdot \boldsymbol{Q}}$	=	Projection of the data onto the shutter waveform for phase-sensitive detection [unitless]

The photocurrents from the three photodiodes (denoted by UV, VIS, and IR in the text) in each spectrometer channel of SIM (Harder et al., 2005b, Section 4.2) are measured with a transimpedance amplifier and digitized by a 16-bit sampling analog-to-digital converter (ADC). The photocurrent is converted to radiant power by replacing $\alpha (\lambda ,T,t)$ in Equation 2 with the photodiode’s radiant sensitivity [$R_{\mathrm{S}}(\lambda ,T,t)$] in units of Amperes Watt^−1^. Equation 4 shows the detector photocurrent; however, the temperature and time dependence derive from changes in the radiant sensitivity found in Equation 2:

4 $$ P_{\mathrm{diode}} \left ( \lambda ,t \right ) = \frac{V_{\max} M_{\mathrm{diode}}^{-1} \left ( D \left ( t \right ) - D_{0} \right )}{G_{\mathrm{amp}}} \frac{1}{R_{S} \left ( \lambda ,t \right )}, $$

**Table:** 

*V*_max_	=	±10 Volts
*M*_diode_	=	65,536 data numbers
*D*(*t*)	=	Data numbers with shutter open
$D_{0}$	=	Data numbers with the shutter closed
*G*_amp_	=	Transimpedance amplifier gain [Ohms]
$R_{\mathrm{S}}(\lambda ,T,t)$	=	Photodiode radiant sensitivity [Amperes Watt^−1^]

Once on-orbit, the pre-flight calibration must be maintained by corrections to key components in the chain of optical elements, detectors, and electronic subsystems that contribute to the parameters in Equations 2, 3, and 4. Equation 3 identifies the ESR closed-loop gain [$\tilde{G} \left ( t \right ) $] as a clock-time-dependent function. To track this time dependence, routine measurements of the gain are made throughout the mission (Harder et al., 2005b, Section 4.1.1) and are found to be very stable with time, with a degradation of about 7 parts per million (ppm) per year, and are routinely corrected in data processing. The most plausible explanation of this kind of degradation is continuous bombardment by energetic particles, predominantly protons from the space environment, that tends to affect leakage currents in amplifiers over time, thereby potentially changing the gain and offset characteristics of critical analog circuitry.

Changes in the photodiode radiant sensitivity show wavelength, time, and temperature dependencies, both listed as accumulative and non-accumulative effects in Table 1. Orbital changes in spacecraft temperature must be continuously corrected, and the long-term trend in the value of $R_{\mathrm{S}}$ must be tracked separately. Energetic-particle bombardment is also responsible for this long-term trend. Sections 4.3 and 6 discuss these two effects.

### Prism-Transmission Degradation

As noted in Table 1, prism-transmission degradation is the leading cause of irreversible loss of instrument response. Detailed discussion of Equation 5 is deferred to Section 7. The important point to note about Equation 5 is that two interlinked phenomena are observed in the time series of SIMA and SIMB. The prism-degradation function [$pd$_AB_$(\lambda ,t_{\text{exp}},W)$] shows that the actual value of the equation differs according to which spectrometer is targeted (designated by subscript AB) and which detector is being used (designated by variable $W$). SIMA and SIMB are exposed to solar radiation at different rates, so SIMB has about a factor of four less exposure to damaging ultraviolet radiation. Exposure times for both spectrometer channels are acquired from telemetry of the entrance-slit shutter state and spacecraft planning and scheduling during telemetry blackouts. Equation 5 basically shows the addition of two terms with a pre-exponential component and an exponential term. The pre-exponential part accounts for the contribution of refraction geometry to degradation, and the exponential part accounts for the opacity of an absorbing layer of material adhered to the face of the prism with a thickness growing with the length of time that the instrument is exposed to solar radiation (exposure time: $t$_exp_). The relative rate of exposure between SIMA and SIMB is not uniform in time, so a relative rate coefficient is needed to account for short-term changes in exposure; this is designated as $f '(\lambda ,t)$ in Equation 5. This exposure correction [$f '(\lambda ,t)$] was initially just unity for the early SIM degradation corrections, but it became clear with the long SORCE mission that there is also an additional correction needed for degradation as a function of clock time (not solar exposure related). In the exponential term of Equation 5 both the absorption coefficient [$\kappa (\lambda )$] and the relative rate contribution [$f '(\lambda ,t)$] are common to both the SIMA and SIMB channels, the only difference is due to $t$_exp_.

$$ pd_{\mathrm{AB}} \left ( \lambda , t_{\exp},W \right ) = \left ( 1- a_{W} \left ( \lambda \right ) \right ) \mathrm{e}^{\left ( -\kappa \left ( \lambda \right ) t_{\exp} f ' \left ( \lambda ,t \right ) \right )} + $$

5 $$ \left ( a_{W} \left ( \lambda \right ) \right ) \mathrm{e}^{\left ( \frac{-\kappa \left ( \lambda \right ) \mathrm{t}_{\exp} f ' \left ( \lambda ,t \right )}{2} \right ) \omega \mathrm{t}}, $$

**Table:** 

*λ*	=	Wavelength [nm]
$t_{\text{exp}}$	=	Exposure time [seconds], different for SIMA and SIMB
*t*	=	Clock time [seconds]
*W*	=	Detector designation (ESR, UV, VIS1, IR)
$a_{W}$	=	Inflight ray-path parameter for detector *W*
*κ*(*λ*)	=	Absorption coefficient of absorbing film
$f ' (\lambda ,t)$	=	Degradation-rate coefficient

Equations 2 – 4 provide the detailed equations for the measurement of spectral irradiance for the ESR and photodiodes, and Equation 5 provides the correction for prism transmission that is a function of clock time, exposure time, wavelength, and detector for each of the two spectrometer channels. Since prism degradation increases with time, the final corrected spectral irradiance is found by dividing by the prism-degradation function [$pd$_AB_]; lower degradation values correspond to a value closer to unity. This is shown in Equation 6:

6 $$ E= \frac{P}{S} \frac{1}{pd}. $$

Here, we suppress functional arguments expressed in Equations 2 through 5, since they are different for each spectrometer channel and detector.

Aside from the detector and prism-transmission contributions to Equation 6, two additional contributions must be included: The 1-AU correction and the Doppler shift for the SORCE spacecraft are determined from three line elements (TLE) supplied by NORAD (www.celestrak.com/NORAD/elements/).

## Impact of Spacecraft Performance on Degradation Corrections

The SORCE spacecraft power, attitude, and electronic systems were designed for a five-year mission, hence the level of critical component reliability and redundancy was specified accordingly. The SORCE spacecraft readily passed all its designed lifetime requirements for a five-year mission; however, in the extended mission, particularly after 2009, corrections to subsystem aging became an increasingly critical part of maintaining spacecraft and instrument health. Of particular importance for the performance of the SIM instrument were the following events:

i) A series of spacecraft safe-hold events changed the optical alignment of the CCD steering mirror (see Section 5). Up until October 2009 these safe-hold events were caused by a software fault on the spacecraft onboard computer (OBC) that caused the spacecraft to revert to a safe-hold state, turn off the instrument power, and activate survival heaters that maintained instrument temperatures at about −40 °C. This OBC software fault was corrected in October 2009 and subsequent safe-hold events were related to battery-cell failures (see item 4 below).
ii) In April 2008, a reaction-wheel anomaly produced lower-quality pointing data for about one week. The operations team turned off the high-friction reaction wheel and installed flight software with a three-wheel control solution that effectively returned the spacecraft back to the same pointing capabilities seen with four-wheel control. Likewise, in September 2012 the power supply for one of the redundant star trackers failed and revised operations with only one star tracker commenced almost immediately with no detectable effect on data quality.
iii) In the first year of the mission, the prism drive was operated out of design specifications causing a wavelength shift in the data – the corrections for this are presented in Section 5.
iv) By September 2010 management of battery power due to the inevitable degradation of the nickel-hydride common pressure vessel (CPV) battery cells dictated every-orbit power cycling first for SIMB starting in September 2010 and then for both SIM instruments starting in May 2011.
v) In July 2013, two additional battery-cell failures caused the battery voltage to drop below the brownout threshold for the primary OBC during eclipse periods. The SORCE Mission Operation team developed a process to manually turn on the OBC on every orbit sunrise (≈16 times per day), then load and execute stored commands to turn on all four of the SORCE instruments during orbit day and off again prior to sunset. This highly complex mission scenario is referred to as the Day Only Operations (DO-Op) mode in this document. As discussed in subsequent sections, daily corrections are made for wavelength shift and temperature drift. Additional analysis is needed for instrument-sensitivity changes during time periods of telemetry blackout where communication with the satellite was disrupted. These disruptions were particularly detrimental to ESR data acquisition resulting in large gaps in the 1600 – 2401.5 nm infrared portion of the spectrum.

**Figure 1:**
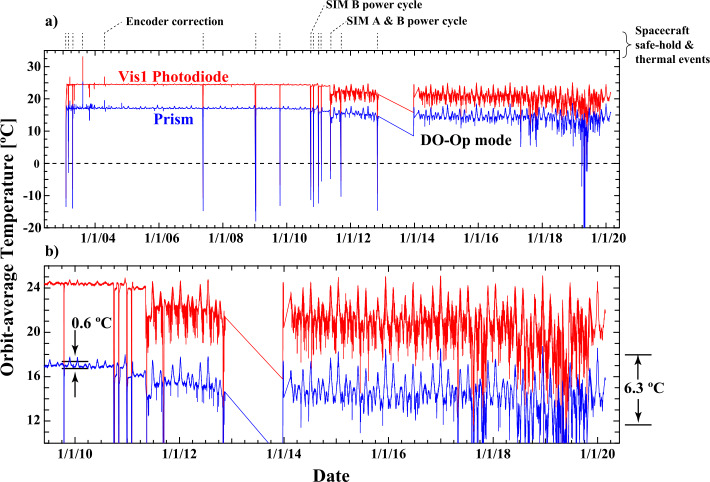
($\mathbf{a}$) The time series for two important telemetry items: the temperature of the VIS1 photodiode and the prism-drive temperature for SIMA. *Dotted lines at the top of the plot* show the times of spacecraft and safe-hold thermal events where changes in the instrument performance occur. Note that these safe-hold events are accompanied by large excursions in temperature. ($\mathbf{b}$) The same data, but over a four-year time period to show more details on the temperature structure. Note that before power cycling, typical temperature variations are on the order of 0.5 – 1.0 °C owing to heating of the whole spacecraft structure, which is due to the changing length of the orbit day. After power cycling, orbit-to-orbit variations are significantly larger ($\approx 6.3$ °C) and multi-year drifts in temperature are present. During the DO-Op mode, multiple safe-hold events occurred that are not shown in the figure.

The effects of spacecraft safe-hold events and then power cycling late in the mission can be readily seen in many standard telemetry items; Figure 1a shows the mission-length time series of two of these telemetry points. The temperatures for the detectors and the prism drive are monitored on a ten-second cadence. A number of these OBC events appear in this time series where the prism and detectors abruptly drop to temperatures below 0 °C. None of the SIM internal monitors are available when the instrument is off, so the exact temperature cannot be determined. It then required three to five days for the mission operations team to return the spacecraft to the normal mode of operation. Under normal operating conditions and up to the onset of power cycling (see Figure 1b), the SORCE spacecraft produced a remarkably stable temperature environment, and this allows for very stable component temperatures within the SIM instrument. Critical component temperatures used in the measurement equation such as the photodiodes, prisms, and ESR are monitored on a ten-second cadence, and data-processing software continuously uses the most recently available temperature data. After the onset of power cycling, the instrument stability drops significantly with orbital variations as large as about 7 °C for the photodiode detectors. The prism temperature shows less orbit-to-orbit variations but shows enhanced sensitivity to semi-annual spacecraft temperature variations associated with the changing spacecraft $\beta$-angle; the angle between the orbit plane and the Sun, and this affects the orbit-eclipse duration. This produces wavelength drift that must be accounted for in data processing and degradation corrections. In the power-cycle mode, the ESR typically does not achieve a stable temperature over the length of the day-lit portion of the orbit, but the phase-sensitive detection method (see Harder et al., 2005b) partially negates this effect. Also seen in Figure 1b is a multi-year drift in all of the temperature monitors that was not present before power cycling. While corrections of temperature-sensitive parameters are made in the SIM data processing, the data quality is degraded more since 2011 when power cycling of SIMA began.

## Short-Term, Non-accumulative Effects

### Perturbations Due to Spacecraft-Pointing Errors

The SORCE spacecraft uses two independent methods of maintaining solar pointing: i) two redundant star trackers are used to maintain satellite orientation, particularly during orbit night, and ii) a quadrant-cell Fine Sun Sensor (FSS) is used to control solar pointing during solar observations and is reported in telemetry every ten seconds. The FSS is hard mounted on the front end of the SIM and the bore sights of the FSS and SIM are collocated to $< 0.5$ arcminutes; thus, the readings from it provide an accurate monitor of off-axis pointing for SIM. The spacecraft reaction-wheel system maintains $0.5 \pm 0.5$ arcminute pointing for SIM throughout most of the orbit. The exception is during spacecraft-roll maneuvers to prevent light contamination from the Earth affecting the operation of the star trackers. Typically, two to four of these roll maneuvers occur each orbit with a different timing with respect to the start of orbit day. Different wavelengths on the different detectors will be affected by this activity. A pointing-error correction was implemented for SIM V27 data processing that corrected the reported irradiance value based on size of the off-axis pointing error and is described in the SIM V27 release notes.

### Scattered Light

Scattered light has a small but non-negligible contribution on the measured spectrum. A nonsequential ray trace of the system indicated a scattered-light contribution of < 5×10^−3^ for the ultraviolet detector focal-plane position and < 10^−4^ for the visible and infrared focal-plane locations. Pre-flight laboratory characterization confirmed these values. Of greater importance is to determine if transmission degradation increases scattered light over the course of the mission. This can be confirmed by analyzing the VIS1 and IR photodiode signals during part of a prism scan where detectable radiation does not impinge on the detector. During the acquisition of the infrared spectrum from 1600 – 2401.5 nm this condition is met, and the shutter is cycled every ten seconds. Scattered light is detectable, and the magnitude of this signal is less than one data number on the ADC, so it is only marginally larger than the noise level of the detectors. This scattered-light measurement was conducted over a nine-year period of the mission, and no detectable change was observed with time. A scattered-light correction was not included in processing and does not appear in the measurement equation.

### Instrument and Detector Temperature Effects

For the SIM photodiodes, the radiant sensitivity (denoted as $R_{\mathrm{S}}$ in Equation 4, with units of Amperes Watt^−1^) has both wavelength dependence and temperature dependence. Figure 12 of Harder et al. (2005b) shows the room-temperature radiant sensitivity for each of the SORCE photodiodes, which can have a range of about an order of magnitude in the 200 – 1600 nm spectral range. In addition to this wavelength dependence, the detectors are also temperature dependent; a change in the temperature changes the number of photoelectrons generated per Watt of radiant power. Equation 7 expresses the rate of change of radiant sensitivity with temperature as:

7 $$ \frac{\mathrm{d} R_{\mathrm{S}} \left ( \lambda \right )}{\mathrm{d} T} = \beta \left ( \lambda \right ) \left ( T- T_{0} \right ). $$

In this equation, $\beta (\lambda )$ is the temperature coefficient of radiant sensitivity with units of inverse temperature [°C^−1^] and $T_{0}$ is the room-temperature reference value. The radiant sensitivity is defined at room temperature, hence Equation 8 then provides the deviation of the radiant sensitivity from the room-temperature value:

8 $$ R_{\mathrm{S}} \left ( \lambda ,T \right ) = R_{\mathrm{S}} \left ( \lambda , T_{0} \right ) + \frac{\mathrm{d} R_{\mathrm{S}} \left ( \lambda \right )}{\mathrm{d} T}. $$

**Figure 2:**
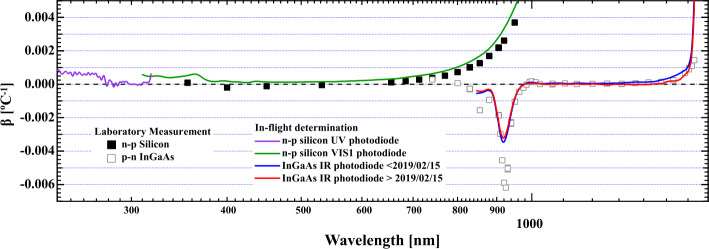
Temperature coefficient of radiant sensitivity [$\beta $] for the inflight SIM n-p silicon and InGaAs photodiodes and a laboratory measurement of flight-spare detectors. The two independent determinations demonstrate excellent agreement except near the margins of detector responsivity. The SIM photodiode data product joins the Si and InGaAs detectors at 950 nm and reports infrared data to 1630 nm. A small revision was made in the long-wavelength portion of the InGaAs photodiode after 15 February 2019 and until the end of the mission.

The temperature coefficient for both the silicon (UV and VIS1) and the InGaAs (IR) detectors was determined on-orbit during the recovery from multiple OBC events and a campaign during the last months of the mission. Figure 2 shows a comparison of the radiant-sensitivity temperature coefficient measured on-orbit compared to a laboratory study of SORCE flight-spare photodiodes performed using the LASP Spectral Irradiance Facility. Agreement between these two independent measurements is excellent; slight variations at the margins of the detectors’ response are expected due to detector-to-detector manufacturing variability, but the comparison validates the on-orbit determination at wavelengths where the detectors are used.

### Detector Noise

The best estimates of the ESR detector noise floor are on the order of 4 nW for a 200-second integration period at a shutter frequency of 0.01 Hz (i.e. 50 seconds open, 50 seconds closed, for two cycles), and the photodiodes are ADC (analog-to-digital converter) limited rather than photon shot-noise limited. The noise for the photodiodes is simply the root-mean-square (RMS) detector photocurrent measured with the shutters closed after the amplifier offset is subtracted. The detector noise power is not affected by such external factors as the increased levels of particle-induced noise as the spacecraft flies through the South Atlantic Anomaly, or semi-annual temperature changes associated with the orbit-eclipse duration.

The photodiode detectors used for this study are not photon-noise limited, but rather ADC-noise limited with a typical RMS ADC noise level of 1.7 bits out of 15 with the same apparent noise level independent of signal strength. The noise equivalent irradiance (NEI) values are determined from this measured noise power for each detector and then converted to irradiance via an integral profile described in Equation 2. This is of great importance to observations of solar-cycle variability because at some wavelengths the NEI becomes comparable in magnitude to the observed solar-spectral-irradiance change. For long-term degradation corrections, how large the trend uncertainties are over a fixed length of time relative to the noise equivalent irradiance (NEI) is the best figure of merit for the quality of the correction.

**Figure 3:**
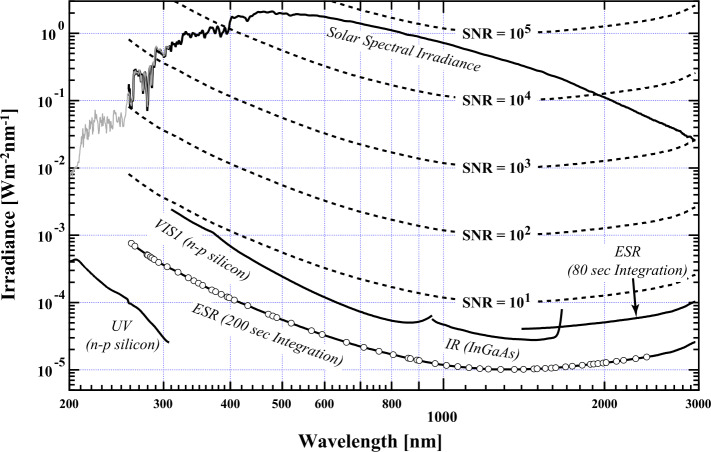
Noise equivalent irradiance (NEI) of the SIM detectors compared to the solar signal level. The plot shows contours of SNR relative to the ESR measurement with a 200-second integration time, indicating that the solar signal is measured with a value more than $10^{3}$ for most wavelengths. The *discrete points* indicated on the ESR noise curve are the 61 points measured throughout the mission. The ESR 80-second integration mode is used to acquire the 1600 – 2401.5 nm data product beyond the sensitivity range of the infrared photodiode. The curves for the ESR extend to 3000 nm, but they only have sufficient SNR to 2400 nm.

Figure 3 shows the NEI of the detectors relative to the solar signal represented by the ESR for the visible and infrared portions of the spectrum and the UV photodiode overlapping with the ESR measurement in the 260 – 308 nm region. Figure 3 also shows contours of the signal-to-noise ratio (SNR) relative to the ESR operating in phase-sensitive detection mode at 0.01 Hz for two cycles (a 200-second observation). These contours indicate that for the visible wavelengths ($\lambda > 400$ nm) the SNR is well in excess of 10^3^; this level of precision is mandatory for measuring solar variability but remains somewhat marginal for the least variable wavelengths in the mid-visible and long-wavelength infrared spectral regions and the low-signal UV shortward than 240 nm. The 310 – 365 nm range for the VIS1 photodiode has a SNR ratio less than 1000 and drops to about 230 at 310 nm.

## Wavelength Shifts

Repetitive modulation of the prism temperature due to changes in the spacecraft $\beta$-angle appears as modulation of the irradiance signal. This is readily corrected in routine data processing through the temperature coefficient of the index of refraction of fused silica reported by Malitson (1965). Appendix A of Harder et al. (2005b) describes the equation set for converting from prism-rotation angle to refraction angle and wavelength. Of greater consequence are the changes induced by the safe-hold events, where a distinct shift in the level of the photocurrent appears after these events. As discussed by Harder et al. (2005a), the rotation angle of the prism is measured using a separate light path above the prism that reflects off a spherical folding mirror and is imaged as a 0.1-mm wide light spot on a 16,000-element linear CCD mounted near the instrument detector focal plane. As the prism is rotated, the image spot moves across the CCD providing a highly accurate measurement of the prism-rotation angle. A separate spectrometer light path illuminates detectors in the focal plane. The prism and folding mirror are hard mounted and corotated with a fixed relative orientation. The control of the prism rotation angle is determined by the position of the light spot on the CCD. The folding mirror is mounted in a pocket using Cu–Be springs that press the glass mirror against three brass pads that are shimmed to aim the outgoing light beam at the CCD. Best indications are that with a 30 C temperature change during one of these safe-hold events, the folding mirror in the CCD optical chain relaxes into a slightly different position. The image spot on the CCD greatly overfills the vertical direction so a shift in this direction will not affect operation, but a shift in the rotation plane of the instrument will induce a wavelength shift.

Correction for the wavelength shift is accomplished by variation of the measured instrument constants related to the prism-dispersion equation rather than through an arbitrarily assumed polynomial relation. These dispersion equations are discussed in Appendix A of Harder et al. (2005b), and the algorithm is described in detail in Section 5.1 of the ATBD (Harder et al., 2021). All of the quantities for the shift-stretch algorithm are determined through pre-flight metrology and then further refined by comparison with other high-quality solar reference spectra. The 21 April 2004 SIM solar reference spectrum presented by Harder et al. (2010) is used as the wavelength standard for all subsequent wavelength corrections throughout the mission.

Over the full mission and after the prism drive has settled into a stable configuration after the safe-hold events, the standard deviation in the retrieved pixel values from one spectrum to the next is on the order of 0.36 sub-pixels. A pixel refers to the 6.5-μm CCD pixels, and onboard hardware subdivides a pixel providing a one-fifth pixel image centroid, which translates to a 0.67 arcsecond per CCD sub-pixel step as measured in the instrument focal plane. Since the optical system for the spectrometer nominally images the entrance slit onto the exit slit (up to image aberrations), the full-width half-maximum (FWHM) of the instrument profile in units of sub-pixels is 232 sub-pixels (equivalent to the 300-μm entrance slit width). The instrument’s resolution is sampled with 38 sub-pixel steps. The uncertainty in the wavelength scale is about $1.5\times 10^{-3}$ of the instrument’s wavelength-dependent resolution. As an example, at 320 nm the FWHM resolution of SIM is 1.65 nm, so the spectrum-to-spectrum wavelength scale is stable to $\approx 2.6\times 10^{-3}$ nm or $\Delta \lambda $/$\lambda \approx 8\times 10^{-6}$. As the width of a resolution element increases at longer wavelengths, the wavelength uncertainty becomes proportionally larger. Harder et al. (2005a) show the instrument’s resolution for the ESR over its operating range. The resolution varies from 0.579 nm at 242 nm to a maximum of 34.59 nm at 1283 nm, hence wavelength uncertainty at these two wavelengths would be 0.0008 nm and 0.052 nm, respectively.

## Photodiode Degradation

The determination of the overall degradation trend needs to include a contribution for photodiode degradation and be determined separately from the loss of prism transmission. The expectation for Si photodiode degradation is that proton bombardment of the UV and VIS1 photodiodes will decrease their radiant sensitivity relative to the ESR. Here, radiant sensitivity is defined as the photocurrent generated by the photodiode per watt of incoming radiation at a given wavelength. The mechanism for photodiode sensitivity loss tends to occur in the base region of the diode and has the effect of decreasing the minority-carrier diffusion length, thereby effectively moving the location of the peak of quantum efficiency to shorter wavelengths with a concurrent decrease in quantum efficiency at longer wavelengths (Baicker and Faughnan, 1962). Quantum efficiency is the number of photoelectrons generated per incoming photon. Since the absorption coefficient of silicon is so large at short wavelengths, the minority-carrier diffusion length for the skin of the photodiode dominates its performance at shorter wavelengths and tends to be less susceptible to this degradation mechanism. Hence, the expectation is that photodiode degradation will be most evident for wavelengths greater than about 750 nm and very weak for the ultraviolet portion of the spectrum.

**Figure 4:**
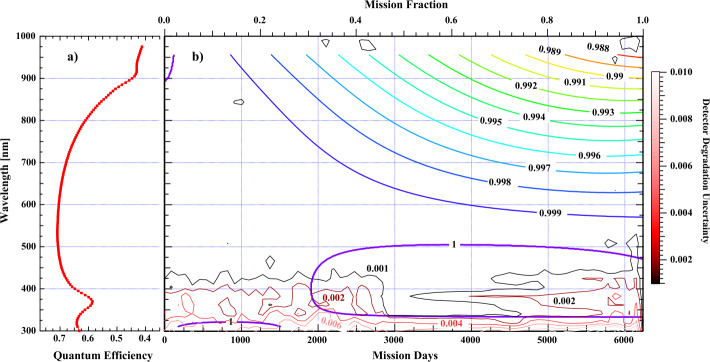
**(a)** The pre-flight quantum efficiency of the VIS1 photodiode for SIM. (**b)** A contour plot showing lines of equal photodiode degradation as a function of wavelength and time. Over the course of the mission, the longer wavelengths degrade faster with time and at 900 nm the diode is degraded about 1% relative to the radiation-hard ESR detector. Uncertainties based on the mutual VIS1 and ESR ratio are shown as *red–black* contours.

Figure 4b shows a contour plot of equal degradation as a function wavelength and time for the VIS1 n-p photodiode relative to the ESR; the bottom and top axes of Figure 4b are mission date and mission fraction extrapolated out to 02 August 2020, past the end of the mission. Figure 4a shows the pre-flight detector quantum efficiency, indicating that the photodiode degradation increases in magnitude and rate with decreasing quantum efficiency, in agreement with the Baicker and Faughnan study. As an example of the rate of photodiode degradation, at 900 nm about 28% of the mission elapsed before a photodiode degradation trend of 0.2% relative to the ESR could be detected, whereas ≈ 90% of the mission elapsed before the same level of degradation could be detected near the peak of the quantum-efficiency curve near 625 nm.

## Prism Degradation

### Basic Concepts of Single-Surface Degradation

By design, the ESR is the least susceptible component in the instrument to radiation damage and is the most stable component in the system over the course of the mission. Light detection for the ESR is through temperature control of two bolometers with cermet thermistors as the active detector elements. Pre-flight radiation testing is important to assure the hardness of the components against possible damage while in space. The bolometers are located inside a reflective hemisphere that minimizes common-mode temperature differences, protects them against further proton and atomic-particle bombardment, and improves the effective blackness of the bolometer through multiple reflections (Harder et al., 2005a). There is the potential for a contamination effect due to loss of reflectivity in the optical sphere that surrounds the ESR. As photochemically active radiation is already filtered out, this mechanism is unlikely to contribute more than a 1 – 2% change on the reflectivity of the sphere. The sphere only contributes 1 – 3% of the total signal of the detector, so there is at most a ≈1 – $6\times 10^{-4}$ contribution due to this possible degradation mechanism. We therefore use the ESR as the reference detector to characterize degradation of other system components in SIM.

The starting point for deriving the prism-degradation function is to analyze the special case where the only source of signal loss is through light attenuation of an absorbing material adhered over time onto the surface of the prism. In this case, the degradation can be expressed through Lambert’s Law as a function of wavelength [$\lambda $], at two different clock times [$t_{1}$ and $t_{2}$], for the A and B spectrometers:

9 $$\begin{aligned} \begin{aligned}[b] &\ln \left ( I_{A} \left ( \lambda , t_{1} \right ) \right ) = \ln \left ( E \left ( \lambda , t_{1} \right ) \right ) - \tau _{A} \left ( \lambda , t_{1} \right ), \ln \left ( I_{B} \left ( \lambda , t_{1} \right ) \right ) = \ln \left ( E \left ( \lambda , t_{1} \right ) \right ) - \tau _{B} \left ( \lambda , t_{1} \right ) \\ &\ln \left ( I_{A} \left ( \lambda , t_{2} \right ) \right ) = \ln \left ( E \left ( \lambda , t_{2} \right ) \right ) - \tau _{A} \left ( \lambda , t_{2} \right ), \ln \left ( I_{B} \left ( \lambda , t_{2} \right ) \right ) = \ln \left ( E \left ( \lambda , t_{2} \right ) \right ) - \tau _{B} \left ( \lambda , t_{2} \right ). \end{aligned} \end{aligned}$$

In Equation 9, $E$ is the irradiance of the Sun, $I$ is the light intensity recorded by the detector(s), and $\tau $ is the optical depth of the material adhered to the face of the prism. These simultaneous equations can be combined, and $E$ can be eliminated to give Equation 10:

10 $$ \ln \left ( \frac{\left ( \frac{I_{A} \left ( \lambda , t_{1} \right )}{I_{A} \left ( \lambda , t_{2} \right )} \right )}{\left ( \frac{I_{B} \left ( \lambda , t_{1} \right )}{I_{B} \left ( \lambda , t_{2} \right )} \right )} \right ) = \Delta \tau _{B} \left ( \lambda , t_{1} - t_{2} \right ) - \Delta \tau _{A} \left ( \lambda , t_{1} - t_{2} \right ). $$

Equation 10 provides a procedure to determine degradation through the “ratio of a ratio”. There are a number of advantages to this approach because the method does not rely on having completely matched absolute calibrations for the two spectrometer channels, because ratios of SIMA as a function of time and SIMB as a function of time remove this dependence. On the days where SIMA and SIMB are compared, differences in the start of the scans were no more than two seconds, so time differences in solar irradiance between the two channels does not enter into the determination. The time-dependent ratio of the A and B channels gives the difference in the optical depth between the two channels. As light attenuation increases with the length of time that a component is exposed to solar radiation, the prism is apparently coated with a material of growing thickness over time.

**Figure 5:**
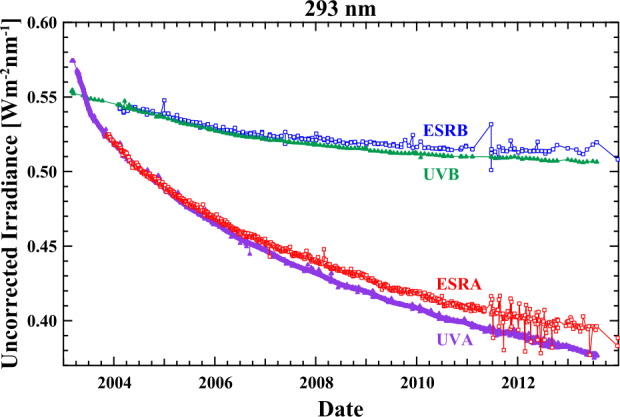
A time series of uncorrected irradiance at 293 nm for SIMA and SIMB. Both exponential-like signal decay and differences in the decay rate appear between detectors in the same SIM channel. The prism-degradation function must be able to reconcile these different behaviors.

While the simplicity of Equation 10 is appealing and provides a starting point for the determination of the degradation function, a number of additional considerations are required to account for the actual measured quantity. Figure 5 shows uncorrected SIM data for both the SIMA and SIMB channels for the ESR detector and the UV photodiode at 293 nm prior to the start of the DO-Op mode. A number of important modifications need to be made to the Lambert Law equation. The amount of degradation apparent in the SIMB channel, measured on a monthly cadence, is significantly smaller than in the daily measurements of the SIMA channel. While there is an approximately exponential decay in the signal strength, no single exponential function accounts for the rate of degradation with time. Furthermore, differences appear between the two detectors that are in the same spectrometer channel. This same observation appears at every wavelength and for each SIM detector, only differing in the magnitude of the degradation. The form of the final degradation function then has to incorporate two important attributes: i) The refraction geometry of the instrument must be included in the prism-degradation function since the detectors are in different locations in the focal plane (Harder et al., 2005b). ii) The degradation has both solar-exposure-time and clock-time dependencies that must be treated separately and combined into a single trend. In the next sections, we describe these additional contributions and show how they influence the form of the prism-transmission degradation function. A detailed analysis of this process in presented in the SORCE/SIM ATBD, and it will be outlined here.

### Ray-Path Contribution (Pre-exponential Part)

**Figure 6:**
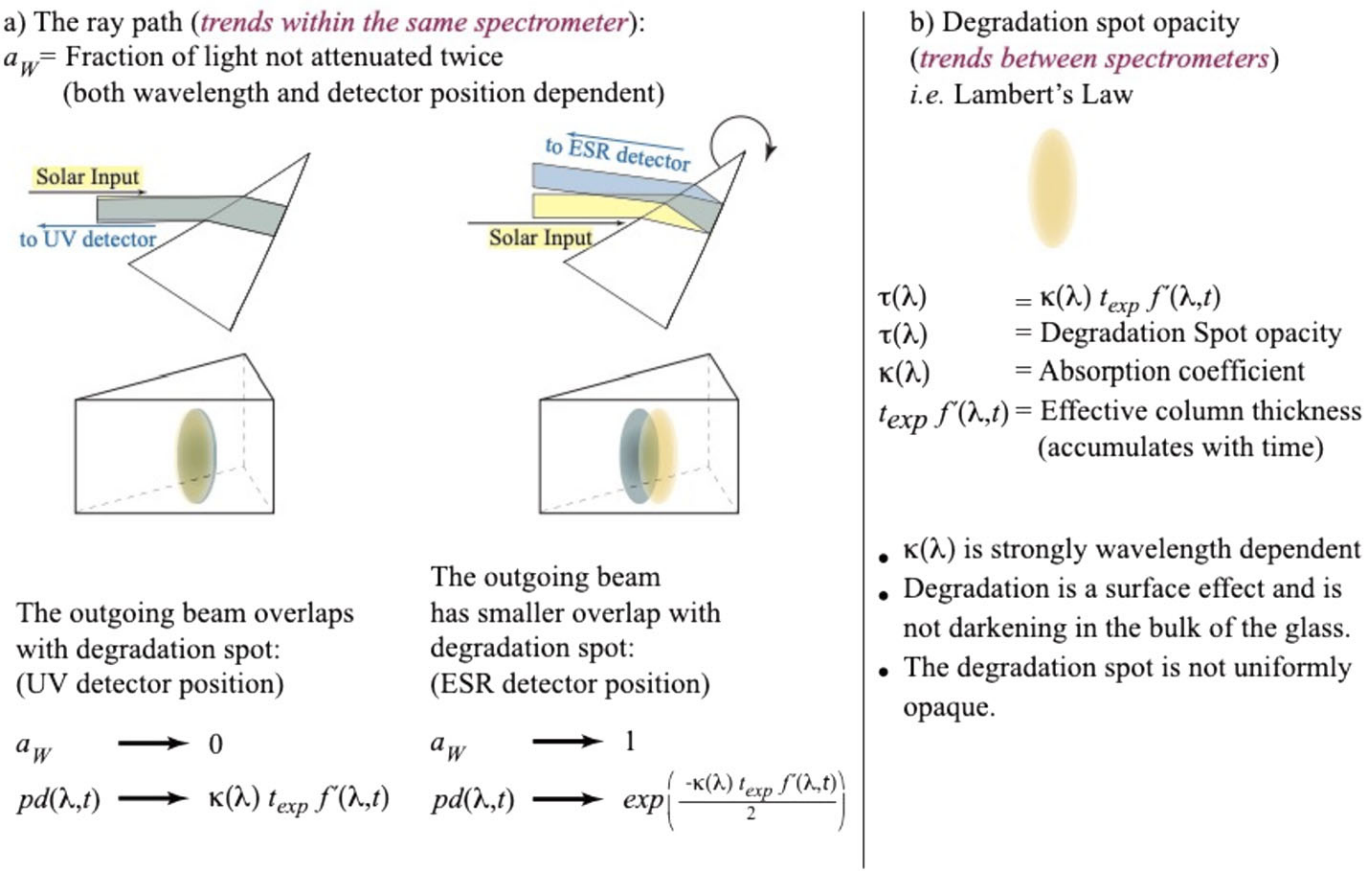
A cartoon depicting the form of the degradation equation (Equation 5). **(a)** The ray-path contribution as the prism is rotated between the two detector positions for the UV photodiode and the ESR. The amount of separation between the two spots is exaggerated for clarity. See Section 7.2 for more details. **(b)** Summary of the characteristics of the opacity of the degrading film adhered to the front face of the prism. See Section 7.3 for more details.

The discussion in Section 7.1 assumes both complete coalignment between the incoming and outgoing light rays that pass through the degradation spot on the face of the prism, and that the degradation spot is uniformly opaque. In reality, this is not true in either case because light refracted and then reflected off the second surface of the prism follows a different light path as it exits the prism on its way to the detectors in the focal plane. The light spot has a non-uniform intensity gradient across its face (i.e. a convolution of a 0.5° solar disk with a rectangular entrance slit) and is further smeared by diffraction and spacecraft-roll maneuvers. The majority of the light rays will encounter this surface film twice, once entering the prism and the other exiting the prism, but a smaller fraction will encounter the central degradation spot only once. In essence, the ray-path parameter [$a_{W}$] is the area of the outgoing light spot on the face of the prism not encountered twice relative to the area of the incoming light spot. This process is depicted in cartoon form in Figure 6a with the incoming light beam shown in yellow and the outgoing beam in blue. The UV detector is nearly in a Littrow configuration and the incoming and outgoing light paths more closely overlap. As the prism is rotated so that light of wavelength $\lambda $ reaches the UV photodiode, $a_{W}$ approaches a value of zero, and the prism-degradation function [$pd(\lambda ,t)$] approaches the full double-pass degradation condition of $pd(\lambda ,t)= \text{e}(-\kappa \, t\exp {f'})$. In the case where there would be complete separation. $a_{W}$ approaches a value of unity and the prism degradation would be $pd(\lambda ,t) = \text{e}^{ ( \frac{- \kappa \, t \exp f'}{2})}$. Note that in Figure 6a the amount of separation between the two spots is exaggerated for clarity, and complete separation in the light spots never occurs. Further refinements in determining the ray-path parameter appear in Section 7.3.1.

### Light Attenuation (Exponential Part)

**Figure 7:**
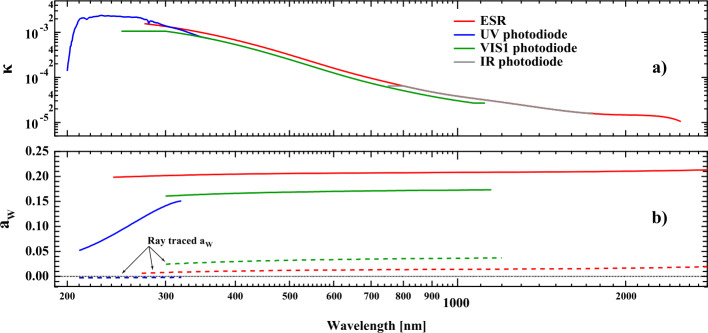
Wavelength-dependent components of the degradation function used in Equation 5. **(a)** The prism-surface film absorption coefficient [$\kappa $($\lambda $)], for the material adhered to the face of the prism. Section 7.3.1 describes how this quantity is derived. **(b)** The ray-path parameter [$a_{{W}}$] for each detector position. The ray-traced initial estimate is shown as a *dashed line*, with the on-orbit values shown as *solid lines*. The ray-path parameter is discussed in Section 7.3.2.

**Figure 8:**
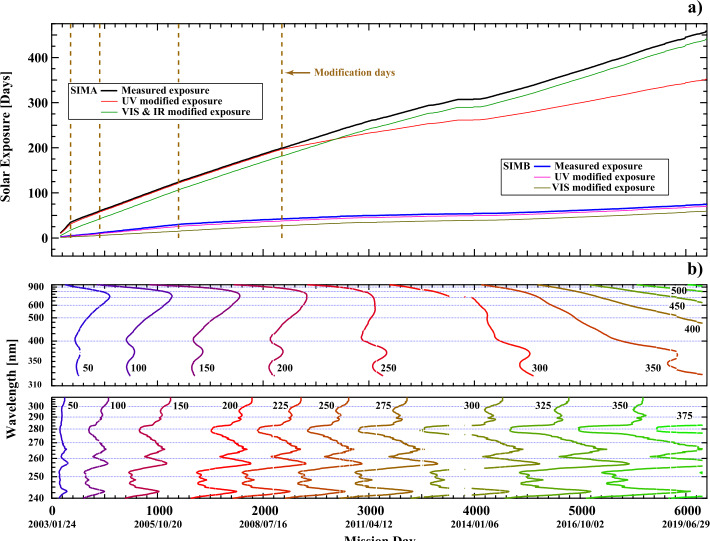
The time-dependent components of the degradation function used in Equation 5. ($\mathbf{a}$) the exposure time for SIMA and SIMB. The lines corresponding to measured exposure time are derived from telemetry and discussed in Section 7.3.3. Section 7.3.5 describes the modification required to the solar exposure for the UV and VIS1 detectors. ($\mathbf{b}$) contour plot of the cumulative column thickness [$C_{\mathrm{a}}(\lambda ,t)$]. The *contours* are derived from a modified solar-exposure record that is accumulated with $f'(\lambda ,t)$ to give the column thickness, as discussed in Section 7.3.4 and Equation 12.

The difference in optical depth [$\tau $($\lambda $)] is shown in Figure 6b and can then be expressed as a wavelength-dependent absorption coefficient [$\kappa $($\lambda $)] and a material thickness that is decomposed into the product of spectrometer channel exposure time [$t$_exp_] and an additional factor that is related to clock time, noted as $f'(\lambda , t)$ in Equation 5. As the mission proceeds, $t$_exp_ and $f'$ can be accumulated to give the time-dependent growth rate of the thickness of the absorbing layer on the prism surface. The column thickness of the absorbing material is calculated separately for the two spectrometer channels: $C_{\mathrm{A}}(t)$ or $C_{\mathrm{B}}(t)$. The two panels of Figure 7 depict the wavelength-dependent parameters of the degradation function. Similarly, Figure 8 then shows the time-dependent contributions to Equation 5. The following five subsections present their derivation.

In Figure 7a the absorption coefficient [$\kappa (\lambda ,t)$] is strongly wavelength dependent with the absorption coefficient about a factor of 157 times larger at 240 nm than at 2000 nm in the infrared. While the exact chemical composition of the absorbing film cannot be determined, it is likely to be surface-assisted aromatic hydrocarbon polymerization. Many electronic packaging and potting processes employ aromatic hydrocarbon materials with ionization and photo-fragmentation processes occurring in the 8 – 10 eV energy range (Eschner and Zimmermann, 2011) making them candidates for the polymerization process.

#### Prism-Surface Film Absorption Coefficient

On about a 90-day cadence the solar spectrum was measured with the ESR from 270 nm to 2401.5 nm with a sampling of three prism steps per resolution element with an 80-second integration time, this measurement mode is referred to as an ESR full scan. The coefficient of absorption of the prism surface film [$\kappa $($\lambda $)] is determined from the stable operating period in April 2004 and November 2007 using the SIMA and SIMB ESR operating in the full-scan mode. The absorption coefficient is determined analogously to Equation 10, but now requires the usage of Equation 5, as shown in Equation 11:

11 $$ \frac{\left ( \frac{I_{\mathrm{A}} \left ( \lambda , t_{1} \right )}{I_{\mathrm{A}} \left ( \lambda , t_{2} \right )} \right )}{\left ( \frac{I_{\mathrm{B}} \left ( \lambda , t_{1} \right )}{I_{\mathrm{B}} \left ( \lambda , t_{2} \right )} \right )} - \frac{\left ( \frac{pd_{\mathrm{B}} \left ( \lambda , t_{2} \right )}{pd_{\mathrm{B}} \left ( \lambda , t_{1} \right )} \right )}{\left ( \frac{pd_{\mathrm{A}} \left ( \lambda , t_{2} \right )}{pd_{\mathrm{A}} \left ( \lambda , t_{1} \right )} \right )} =0. $$

As in Equation 10, the irradiance of the Sun [$E$($\lambda ,t$)] is eliminated for the two spectrometer channels by using the ratio-of-a-ratio approach, and Equation 11 is derived with the $f'(\lambda , t)$ in Equation 5 set to 1.0 for both channels. In this way, the value of $\kappa (\lambda )$ is based on the measured exposure time only as shown in Figure 8 and Section 7.3.3. The best value of $\kappa $($\lambda $) found by using the Nelder–Meade search algorithm (Press et al., 1992), which then corresponds to the degradation per day of solar exposure. Note that Equation 11 would reduce to Equation 10 if there was no ray-path contribution; thus, a numerical method is required to solve Equation 11 when the ray path is included. The ESR Full Scan data are used for evaluating $\kappa (\lambda )$, which provides a comparable wavelength sampling to the photodiode scans. ESR measurements for wavelengths less than 270 nm do not have a sufficient SNR, so this same procedure is repeated with the SIMA and SIMB UV photodiode detectors and matched in the overlap region where ESR data are available. Figure 7a shows the $\kappa (\lambda )$-value obtained by this procedure in the 200 – 2500 nm region for the UV, VIS1, IR, and ESR detectors.

#### Inflight Ray-Path Parameters

The inflight ray-path parameters appear in Figure 7b. As in the determination of $\kappa $($\lambda $), the most stable time period is from April 2004 – November 2007, and this is used to optimize the values of the inflight $a_{{W}}$ for each detector at their nominal wavelengths. The starting value of Kappa-ESR is determined by using a ray-traced determination of $a_{{W}}$ and counting the fraction of rays that fall outside of the incoming light spot. The inflight ESR $a_{{W}}$ at each wavelength is then adjusted from the ray-trace values so that the differences in the degradation-corrected irradiances between ESRA and ESRB are minimized over the specified time range. The updated $a$_ESR_ and the ESR $\kappa $($\lambda $) are used to produce a corrected time-series irradiance from the ESR. The VIS photodiode ray trace [$a$_VIS_] is then optimized by minimizing the differences between the corrected ESR data and the corrected VIS data over the same time period. As the degradation for the infrared photodiode is so small, the value of $a$_ESR_ is used for the IR photodiode.

One of the important characteristics seen in uncorrected UV and VIS1 photodiode data after removal of the solar-distance modulation is a residual annual modulation with wavelength-dependent amplitude. This additional modulation was identified as being the result of the varying apparent diameter of the Sun projected over the degradation spot on the face of the prism as seen from the Earth’s elliptical orbit. Minimization of this residual modulation provides an effective method of identifying the value of the $a_{W}$ for the UV and VIS1 detectors. Analysis shows that $a$_UV_ is 160 times greater than what was estimated from the ray trace. This discrepancy is an indication of the non-uniformity of the degradation spot on the prism face. This determination properly corrects the annual modulation equally well throughout the spectrum and fixes a persistent problem that had existed in the SIM time series in data releases prior to V20. Note that the time period used to determine the ray path is before photodiode degradation becomes an important contribution.

#### Solar-Exposure Record

A very accurate accounting of the length of time that both spectrometer channels of the instrument are exposed to sunlight is a fundamentally important instrument parameter. Critical measurements of the state of the SIM shutters are recorded on a one-second basis. The exposure record consists of the number of seconds of exposure of the two channels for every orbit over the course of the mission. The operations-planning schedule and spacecraft-data logs are used to recover the state of the instrument in the event of missing flight telemetry packets and data loss at the ground stations. Figure 8a shows this measured exposure and is labeled in the legend. The SIMA channel has accumulated a total of 461 days of solar exposure over the course of 6239 days. Over the same period SIMB (the reference channel) accumulated 75 days of exposure. SIMB is exposed at less than one-fifth the rate of SIMA but the exposure rate is not constant throughout the mission, and accounting for these differences is needed for the degradation correction. Early in the mission, observations of the Sun were performed on SIMA every orbit, but excessive degradation was seen in that channel, so in July 2003 the number of SIMA observations was reduced to limit the rate of exposure. The effectiveness of the solar-exposure record as a measure of degradation will be reevaluated in Section 7.3.5 with the discussion of the need for a modified solar exposure.

#### Relative SIMA SIMB Degradation-Rate Coefficient

The $f'(\lambda ,t)$ gives the change in the *relative* *degradation* *rate* of SIMA and SIMB as a function of wavelength and clock time. If the rate of change of prism degradation were strictly proportional to exposure time, the $f'$-function would be identically equal to 1, and this is clearly not the case, as seen in the discussion of Figure 5. The determination of the $f'(\lambda ,t)$ is between two time periods [$t_{1}$ and $t_{2}$] separated by three months. Sections 7.3.1 to 7.3.3 describe the determination of all of the parameters in Equation 5 except for $f '$($\lambda ,t$). The minimization is accomplished using Equation 11 and is the same procedure that is used to determine $\kappa $($\lambda $); however, $f '$($\lambda ,t$) is now determined over a running three-month time step throughout the mission.

The column thickness of the absorbing layer [$C$_AB_($\lambda ,t$)] is constructed by accumulating the product of the every-orbit exposure time [$t$_exp_], and $f '$($\lambda ,t$) is evaluated for that orbit using Equation 10 in units of exposure days.

12 $$ C_{\mathrm{AB}} \left ( \lambda ,t \right ) = \sum t_{\exp} \left ( t \right ) f ' \left ( \lambda ,t \right ). $$

The column thicknesses are shown for SIMA VIS1 and UV as a contour plot in Figure 8b. Note that this column thickness is a three-dimensional surface with time and wavelength dependencies. There are two observations to note about Figure 8b: i) early in the mission the contours are more closely spaced, indicating a greater rate of degradation; ii) much of the contour structure follows the irradiance-intensity structure, particularly for the UV photodiode. For example, the column thickness [$C_{\mathrm{A}}$($\lambda ,t$)] at 280 nm occurs progressively earlier relative to the surrounding wavelengths. For example, the 300-column thickness contour occurs about 600 days earlier than for the nearby 290 nm wavelength. For SIMB UV and VIS1 detectors, the end-of-mission contours on column thickness [$C_{\mathrm{B}}$($\lambda $,$t$)] reached values of 72 and 64, respectively. As seen in Figure 8b, these SIM B column values occurred for SIMA well within the first two years of the mission.

#### Modified Solar Exposure

As the mission progressed, it was clear that using the as-measured solar-exposure record overestimated the amount of degradation in the instrument. As noted in Figure 8a, science operations on both channels of SIM changed at selected times. On 21 July 2003, 21 April 2004, 08 May 2006, and 9 January 2009 spectral acquisition was modified, thereby producing noticeable changes in the rate of exposure. To address this, a correction to the solar-exposure record was applied on these dates. The magnitude of the multiplicative corrections was optimized for two different wavelengths: at 260.0 nm for the UV photodiode and at 407.0 nm for the VIS photodiode and the ESR. The corrections were also determined for SIMA and SIMB and for the VIS1 and UV photodiodes separately. The degradation parameters described above ($\kappa $ and $f'$) were recalculated with the modified solar-exposure record. These new prism-degradation parameters greatly improved the agreement between the SIMA and SIMB channels and also with the TSI when integrated between 240 and 2401.5 nm. Final values for the exposure time for Version 27 are included in Figure 8a, and a comparison of the integrated SIM SSI to the TSI is given in Section 9.

The need for adjusting the solar-exposure record is an indication that prism degradation is the result of hydrocarbon contamination and the surface-assisted second-order kinetics of the polymerization process requiring both ultraviolet radiation and a chemical reagent to produce the degrading film. As the amount of contaminant is slowly depleted through outgassing, the accumulated dosage of solar radiation has a decreased effectiveness in producing the light-attenuating film. In SORCE/SIM V21 – 27, the modified exposure records were used as outlined here. Future research efforts will reconcile the exposure-time estimates to a single contiguous record without resorting to the necessity of applying a step-function response, as performed up to this point in time.

**Figure 9:**
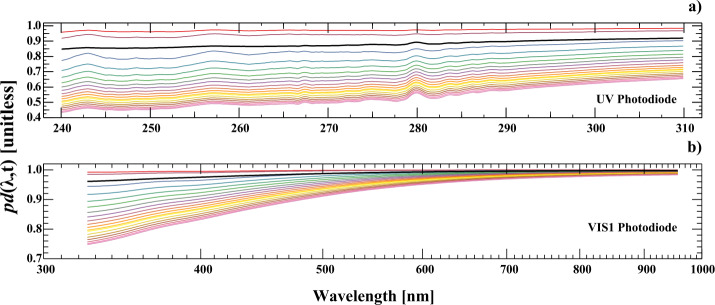
The degradation functions [$pd$($\lambda $,t)] for SIMA for the UV ($\mathbf{a}$) and VIS1 ($\mathbf{b}$) photodiodes are shown as *colored lines* for the full mission on a yearly cadence. The *thick black lines* in these two panels are the final degradation functions for SIMB in February 2020 showing the significantly reduced degradation in SIMB.

The final degradation function is then calculated to produce a valid prism-degradation function for every wavelength and time. Figure 9 shows these functions for the duration of the mission on a one-year cadence for SIMA UV and VIS photodiodes. Also included are the SIMB prism-degradation functions on the last measured day (23 February 2020) to indicate the relative amount of degradation in the two channels. Note that curves in this figure represent an evaluation of Equation 5 with increasing column thickness of absorbing material.

## Time Series and Time-Dependent Uncertainty Estimates

**Figure 10:**
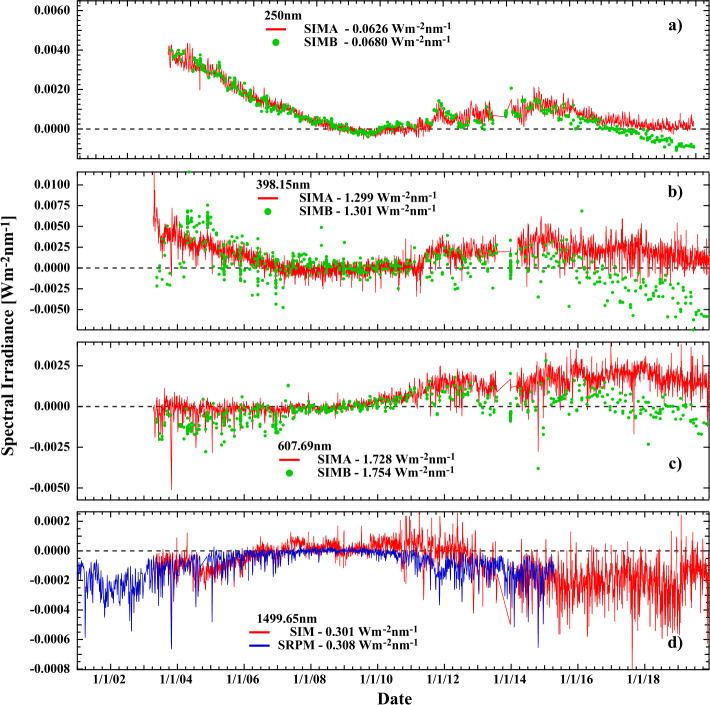
Time series of V27 SIM data at four selected wavelengths. (**a)** – (**c)** show the comparison of the SIMA and SIMB channels with the increased level of disagreement between the two channels after the start of power cycling. ($\mathbf{d}$) compares the SIMA IR photodiode to the Solar Radiation Physical Model (SRPM: Fontenla, Stancil, and Landi, 2015).

Figure 10 shows the corrected irradiance for four different wavelengths throughout the SIM operation range for both SIMA and SIMB. Up until the onset of power cycling for both instruments starting in September of 2011, the mutual agreement between the two SIM channels is very good, but it becomes less reliable after that. Apparent in Figure 10b are slower temperature drifts that further reduce the relative stability of the A and B channels. Referencing Figure 10a, at 250 nm SIM shows a larger irradiance change in the descending phase of Solar Cycle 23 (SC23) than in the rising phase of SC24. Generally, SIM reports a larger change in irradiance than does the SORCE/SOLSTICE instrument and most models of solar variability. In Figure 10b near the Ca ii solar absorption feature, the agreement between the two SIMs is good up until the start of the DO-Op mode. This same situation appears in the visible part of the spectrum (Panel c), but a general change in level is seen between the two channels after the onset of power cycling. Due to the low level of degradation seen in the infrared (Panel d), the anti-solar-cycle trends there are clearly observed, albeit with an increased level of noise in the DO-Op mode. Figure 10d compares the infrared with spectral synthesis data from the Solar Radiation Physical Model (SRPM) (see Harder, Béland, and Snow, 2019). The SIMB IR detector data are not shown in this plot because they did not produce radiometric-quality data due to partial vignetting of the detector. The degradation in the infrared is very small and readily identified as a near-linear slope in the data. Comparisons with the SIMA and SIMB ESR were conducted with the SIMA photodiode detector to validate the degradation correction.

**Figure 11:**
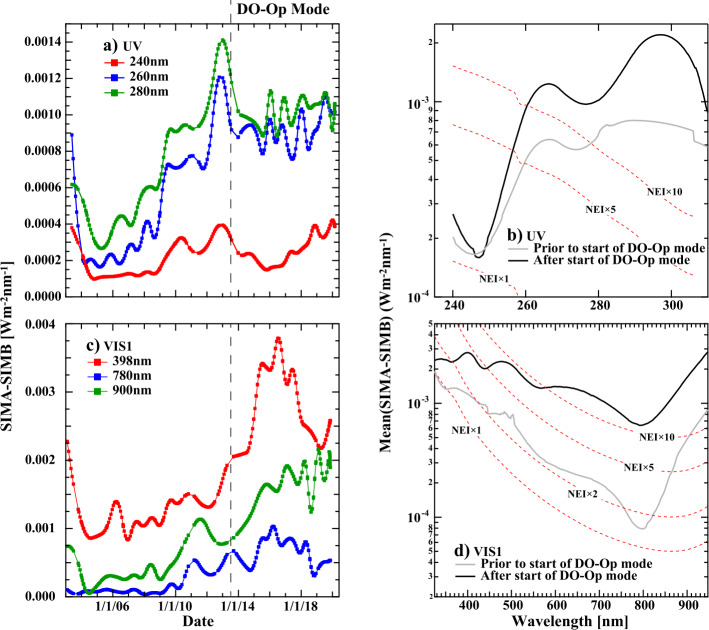
Comparison of SIMA and SIMB. (**a**, **c**) Time series of the differences of time-matched observations between the two instrument channels over the full length of the mission for the UV and visible photodiodes. (**b**, **d**) The mean difference between the two SIM channels as a function of wavelength. Also shown in (**b)** and (**d)** are contours of multiples of the NEI for the detectors indicating the magnitude of the error relative to random detector noise. *Black* traces in (**b)** and (**d)** are the standard deviation of SIMA–SIMB difference after the onset of the DO-Op mode, and *gray* traces show the level of agreement before then. The *gray* trace shows the agreement between SIMA and SIMB during a time period of higher temperature stability.

Figures 11a and c show the absolute value of irradiance difference (units of W m^−2^ nm^−1^) between SIMA and SIMB for 329 matched monthly pairs of observations for three wavelengths in each panel; SIMA and SIMB observations are scheduled to occur simultaneously, so the time stamps differ by no more than two seconds. Concurrent irradiance differences between the two channels are used to determine the stability, or equivalently, the long-term uncertainty of the observation. Since this difference is immune to common mode changes in both short-term and long-term solar variations, any difference between the two channels is instrumental in nature. Deviations from a flat response specify the reduction in measurement stability. Deviations from a flat difference (i.e. a slope near zero as a function of time) between the channels occur early in the mission when spacecraft outgassing occurs, and after 2011 when instrument power cycling started. After 2011, larger deviations occur due to the overall instability in the instrument driven by every-orbit temperature changes (see Section 3) and more frequent safe-hold events that induced mechanical changes in prism orientation, producing discontinuous changes in the instrument response. This effect is most evident in the DO-Op mode for both the UV and VIS1 detectors, where the uncertainties are systematically greater over the full wavelength range.

In the SIM V27 data product, the time and wavelength dependencies are evaluated and reported for every matched SIMA and SIMB pair. Figures 11b and d show average SIM A – SIMB difference before and after the onset of the DO-Op mode for the UV and VIS1 detectors, shown respectively in gray and black traces. To give an estimate of the magnitude of the uncertainty, these panels also show contours of NEI. The noise characteristics are described in Section 4.4. One can see from Figure 11b that the differences are typically less than about $8\times 10^{-4}$ W m^−2^ nm^−1^ for most wavelengths prior to the DO-Op mode, but significantly larger afterwards. Wavelengths less than about 280 nm are less that about a factor of ten lower than the NEI prior to the DO-Op mode. For the visible in Figure 11d, the majority of differences seen between the two SIM channels fall within one – five times the native NEI of the SORCE detectors earlier in the mission, but about a factor of ten or higher after that.

## Integrated SIM Compared to TSI

**Figure 12:**
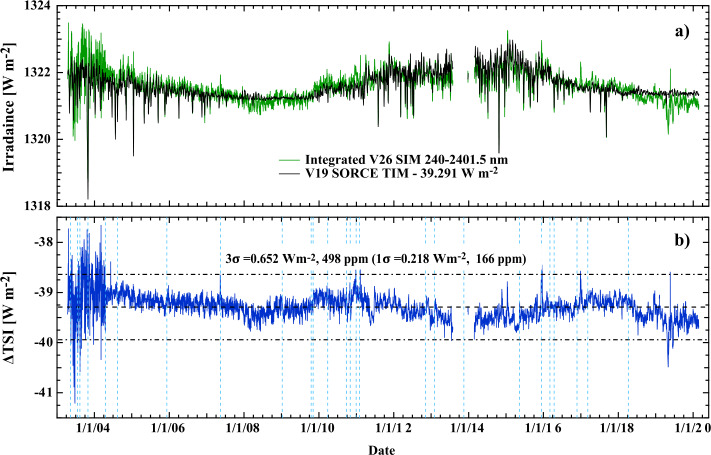
V27 SIM data integrated from 240 – 2401.5 nm relative to the V19 TIM TSI. ($\mathbf{a}$) TSI time series is brought into agreement with the integrated SIM by subtracting 39.291 W m^−2^. (**b)** shows the residual of the subtraction. Shown as *blue-dashed lines* are the locations of safe-hold events. Much of the structure seen in the residuals occurs after these safe-hold events. After accounting for the offset from the TSI, the agreement is about 166 ppm ($k = 1$) relative to the solar-cycle-minimum integrated irradiance.

During an 81-day period of the Solar Cycle 23 minimum, starting on 10 November 2008, V19 SORCE/TIM reported a TSI value of 1350.528 W m^−2^. In this same time period, the SIM spectral irradiance integrated between 240.0 and 2401.5 nm gave a value of 1321.214 W m^−2^, accounting for about 97.8% of the TSI. The comparison with the SORCE/TIM V19 is shown in Figure 12a with the TIM six-hourly data product interpolated to the common scan times of SIM. Over the fulltime range of the mission, Figure 12b shows the average integrated SIM-TIM difference. Over the entire mission, integrated SIM shows an average deficient of 39.291 W m^−2^ from the TSI. The legend for Figure 12b gives the $\pm 1\sigma $ standard deviation of 0.217 W m^−2^; the dashed lines provide the $\pm 3\sigma $ level of 0.652 W m^−2^. Relative to the integrated SIM solar minimum value of 1321.21 W m^−2^, $\pm 1\sigma $ uncertainty corresponds to 166 ppm.

Figure 12b shows a number of important features about instrument performance over the mission. The higher noise levels in the first 450 days of the time series seen in Figure 12b are caused by poor wavelength control for the ESR in the 1598.95 nm to 2401.5 nm range that was corrected after that time period. Conducting the same analysis excluding the first 450 days reduces the standard deviation to 0.207 W m^−2^. In a similar manner, the decrease in the integrated signal after June 2018 is related to temperature effects in the ESR, as noted in Section 3 and shown most clearly in Figure 12b. Over the entire time series, much of the structure seen in Figure 12b occurs at the boundaries of the spacecraft safe-hold events, which are noted as blue-dashed lines. The structure seen in the difference plot is indicative of the magnitude of the overall systematic error in the ability to conduct degradation corrections.

Another method to examine the contribution to errors in the integrated SIM is to perform a trapezoidal-rule integration of the spectrum with a concurrent integral addition of the errors associated with each wavelength step. Each wavelength step has an associated error (Section 5) and every irradiance value has a daily uncertainty (Section 8). Note also that the prism spectrometer has a variable wavelength step, so a trapezoidal-rule integration is required. Performing this trapezoidal integration from 240.0 to 2401.5 nm for the near solar minimum spectrum (10 November 2008) gives an integrated value of 1316.886 W m^−2^ (about 0.3% lower than the more accurate Newton–Cotes integration) and an error on integration of 0.773 W m^−2^, which is about a factor of 3.5 larger than the ±1$\sigma $ standard deviation value of 0.217 W m^−2^. This gives an indication that the errors estimated for the integrated SIM are consistent with the expected best performance of the instrument and indicate that the wavelength and time error estimates discussed in Section 8 are very conservative error estimates.

## Summary and Outlook

It is important to recognize that degradation corrections based on instrument-only corrections, such as those performed on SORCE/SIM, are highly sensitive to a broad range of degradation mechanisms. Objective degradation corrections require designed-in onboard equipment and instrument-operation schemes that are able to account for the myriad sources of signal loss (BenMoussa et al., 2013). This article provides a specific account of the development of the SORCE/SIM degradation-measurement equation and how parameters and uncertainties of this measurement equation are extracted from telemetry, instrument metrology, optical design, and spacecraft performance. Discussion of the mechanism of degradation also highlights that less well-controlled contributions are also very important and must be addressed; in particular, the role of energic-particle bombardment and the photochemical response of residual contaminants must be included. A spaceborne spectral radiometer acts like a photochemical reactor with contaminants being the rate-limiting reagent rather than the length of time it is exposed to solar photons. There is a need to account for both exposure time and clock time to track the ever-changing rate of prism-transmission degradation. The next generation of the SIM spectral radiometer, TSIS-1/SIM now deployed on the *International Space Station*, started observations on 14 March 2018 with several instrument-design enhancements based on lessons learned from SORCE/SIM that will provide more effective degradations corrections (Richard et al., 2020; Mauceri et al., 2020; Harder et al., 2022). Foremost among the SORCE/SIM lessons learned for the TSIS-1/SIM are:

i) An ultrahigh vacuum-compatible optical cavity that completely excludes polymerics;
ii) Strict, non-varying rates of exposure;
iii) Improved detector-noise characteristics;
iv) The addition of a third spectrometer channel to account for degradation in the monthly calibration channel.

The results of the present study have implications both for solar-irradiance modeling and future spectral-irradiance observations. The anti-solar-cycle trends in the descending phase of Solar Cycle 23 reported in the SORCE/SIM instrument (Harder et al., 2009) have been questioned on the basis of potential uncorrected degradation not observed in other spacecraft-borne sensors operable at visible wavelengths (Wehrli, Schmutz, and Shapiro., 2013; Cessateur et al., 2016; Meftah et al., 2016). Furthermore, in the absence of instrument-only corrections, ad-hoc corrections based on curve fitting (Wehrli, Schmutz, and Shapiro, 2013), level shifting (Woods et al., 2018), imposing agreement with the TSI (Mauceri et al., 2018), and reliance on linear scaling through solar minimum (Marchenko and DeLand, 2014) cannot objectively discriminate between anti-solar-cycle trends and some arbitrarily specified notion of degradation. In a similar manner, solar models based on proxies, semi-empirical models, or hybrid versions have dependencies based on the associated details of spectral and temporal coverage with none of them, understandably, universal in scope. Current semi-empirical models such as SRPM (Fontenla, Stancil, and Landi, 2015) and COSI (Shapiro et al., 2010; Ermolli et al., 2013) produce anti-solar-cycle trends in the visible spectrum; however, other efforts such as the Osservatorio Astronomico di Roma (OAR) model (Ermolli, Criscuoli, and Giorgi, 2011) and the Spectral And Total Irradiance REconstruction for the Satellite era (SATIRE-S: Yeo et al., 2014) do not. Similarly, such proxy models as the NRL-TSI/SSI Version 1 and 2 (Coddington et al., 2016) and the EMPirical Irradiance REconstruction (EMPIRE: Yeo, Krivova, and Solanki, 2017) also do not. The increase in solar-disk area of dark structures with the magnetic activity qualitatively supports the conclusions of Foukal (2015) that during cycles of intense activity the TSI variability (hence, also the SSI variability in visible and infrared) might be overestimated by reconstruction models that do not properly consider the existence and contribution of such dark features (Topka, Tarbell, and Title, 1992, 1997).

## Data Availability

The SORCE/SIM data used for this article are Version 27 of SIM and available both through LASP and the NASA Goddard Earth Sciences Data and Information Services Center (GES DISC): lasp.colorado.edu/home/sorce/data/ disc.gsfc.nasa.gov/datasets?keywords=SOR3SIMD_027 Important discussion also appears in the Version 27 release notes found at: lasp.colorado.edu/home/sorce/instruments/sim/sorce-sim-data-products-release-notes/
